# Introduction of Egg White and Yolk to Infant Diets and Early Childhood Atopic Dermatitis

**DOI:** 10.3390/nu15061379

**Published:** 2023-03-13

**Authors:** Man-Chin Hua, Tsung-Chieh Yao, Sui-Ling Liao, Ming-Han Tsai, Shen-Hao Lai, Li-Chen Chen, Kuan-Wen Su, Chih-Yung Chiu, Kuo-Wei Yeh, Jing-Long Huang

**Affiliations:** 1 Department of Pediatrics, Chang Gung Memorial Hospital, Keelung 204, Taiwan; 2 College of Medicine, Chang Gung University, Taoyuan 333, Taiwan; 3 Division of Allergy, Asthma and Rheumatology, Department of Pediatrics, Chang Gung Memorial Hospital, Taoyuan 333, Taiwan; 4 Division of Chest, Department of Pediatrics, Chang Gung Memorial Hospital, Taoyuan 333, Taiwan; 5 Department of Pediatrics, Municipal TuCheng Hospital, Chang Gung Memorial Hospital, New Taipei City 236, Taiwan

**Keywords:** atopic dermatitis, children, egg white, egg yolk, food sensitization, parental allergy

## Abstract

This study investigated whether the introduction of allergenic foods in infancy is associated with atopic dermatitis (AD) in early childhood. Information regarding parental allergic histories, the introduction of six possible allergenic foods (fruits, egg white, egg yolk, fish, shellfish, and peanuts), and physician-diagnosed AD was obtained using age-specific questionnaires (0–2 years). Immunoglobulin E, specific to 20 food allergens, was also quantified at 12 months of age. Logistic regression analyses were used to determine the association between individual food introduction and the outcomes of food sensitization and AD. We found AD development by 2 years of age was significantly related to a parental history of allergy (adjusted odds ratio (aOR) = 1.29) and not being introduced to egg white and yolk during infancy (aORs = 2.27 and 1.97, respectively). Stratified analyses revealed that the introduction of both egg white and yolk was negatively associated with AD by 2 years of age, especially for those children where both parents had allergic diseases (aOR = 0.10). In summary, the introduction of egg white and yolk to an infant’s diet may be a modifiable factor in reducing the risk of physician-diagnosed AD by 2 years of age, which may be particularly important for infants where both parents have allergies.

## 1. Introduction 

Atopic dermatitis (AD) is often the first manifestation of atopy in early childhood and is highly associated with food sensitization and food allergies [1,2]. Young children with AD frequently show sensitization to eggs, milk, or peanuts [2]. Recently, there has been increasing evidence that exposure to food allergens in the environment causes sensitization and food allergies through the disruption of the skin barrier [3,4,5], causing T-cell deviation toward a Th2 response. Meanwhile, early oral exposure may cause T-cell deviation toward the tolerogenic Th1 and T regulatory subtypes through the process of oral tolerance [3]. Clinical studies have also reported that there is no relationship between the age at complementary food introduction and the risk of developing food allergy and AD [6,7], whereas delayed exposure to allergenic foods may increase the likelihood of developing allergies [8,9]. Currently, international guidelines recommend that complementary foods, including allergenic ones, should be introduced to infant diets at around 4–6 months, regardless of whether the infants are at risk of allergies [8,10,11,12]. Among allergenic foods, growing evidence indicates that the introduction of eggs and peanuts to infant diets tends to reduce the incidence of egg allergy and peanut allergy, respectively [7,8,13]. Nevertheless, studies have reported inconsistent results regarding the allergy preventive effects of egg or peanut consumption and AD development later in life [7,13]. 

Cultural traditions and environmental factors may influence the type of infant food or complementary feeding process [14,15]. Compared to many Western countries, food allergies are less prevalent in Asia [15]. Previous studies have also demonstrated that the types of food allergy and sensitization differ in order of relevance in Eastern populations, comparable to those reported in Western populations [15,16,17]. To date, limited studies have evaluated the relationship between the introduction of allergenic foods and the risk of developing food sensitization and AD in Asian children [7,13].

In this prospective cohort study, we aimed to compare the infant feeding practices between children with AD and healthy controls and assessed whether parental allergic histories influenced their dietary choice. We also examined whether the introduction of allergenic foods by the age of 1 year is associated with immunoglobulin E (IgE)-mediated food sensitization at 12 months of age and the occurrence of AD by the age of 2 years between children with and without parental allergic histories.

## 2. Materials and Methods 

### 2.1. Study Participants

A total of 896 children who were delivered at Chang Gung Memorial Hospital, Keelung, Taiwan, between March 2012 and April 2017 were enrolled in the Prediction of Allergies in Taiwanese Children cohort study after obtaining written informed consent from their parents soon after birth [18,19]. To avoid potential confounding factors, 52 infants who dropped out during the 2-year follow-up period; 128 infants who did not regularly return for follow-up visits to the clinic or whose parents did not complete the detailed dietary questionnaires; and 73 infants who had a gestational age of <35 weeks, any perinatal insult, or a major congenital anomaly, were excluded. Furthermore, 57 AD children had relapsing skin rashes before the age of 6 months, and 85 children who never had AD but had allergic rhinitis or asthma diagnosed at the age of 2 years (allergic rhinitis, 73; asthma, 21) were excluded from the analysis. Finally, 501 infants were included. This study was approved by the Ethics Committee of Chang Gung Memory Hospital (202102580B0A3, 201901820A3, 103-6519A3, 100-0225B)

### 2.2. Questionnaires 

All the participants returned regularly for follow-up clinic visits and were checked by pediatricians for general health and atopic manifestations at 2, 4, 6, 12, 18, and 24 months of age [18,19]. Children were diagnosed with AD if they presented with relapsing itchy skin rashes on the face and/or extensors in infancy or on the flexors (e.g., elbows, wrists, and back of knees) and/or creases in the toddler years. After the evaluation, age-specific questionnaires were administered to the parents. Detailed information regarding demographic data; parental allergic history; environmental risk factors; relapsing skin rashes; physician-diagnosed atopic manifestations; current feeding regimens (e.g., human milk, infant formula, or mixed); timing of the introduction of solid foods; and potentially allergenic foods introduced into their diet, such as (1) cereals, (2) meat, (3) fruits, (4) egg white, (5) egg yolk, (6) fish, (7) shellfish, and (8) peanuts were collected prospectively. 

### 2.3. Determination of Serum Allergen-Specific IgE 

Blood samples (3–5 mL) were collected from each subject at 12 months of age. Allergen-specific IgE was determined in 310 participants using an automated microfluidic-based multiplexed immunoassay system (BioIC Allergen-specific IgE Detection Kit-AD40 Panel; Agnitio Science and Technology, Hsinchu, Taiwan) [18]. Twenty food allergens, including cow’s milk, goat’s milk, egg white, egg yolk, crab, shrimp, codfish, salmon, blue mussel, soybean, wheat, potato, peanut, almond, garlic, cheese, baker’s yeast, kiwi, tomato, and carrot, were screened. Those participants who were sensitized to one or more food allergens as determined by BioIC (class score 1–6) at the age of 1 year, were defined as having food sensitization.

### 2.4. Definitions Used in the Study

Children who developed atopic dermatitis by 2 years of age (AD children): Children who had relapsing skin rashes that had been confirmed as AD by a physician by the age of 2. 

Non-allergic children: Children who had not been confirmed to have allergic diseases, such as AD, allergic rhinitis, or asthma, and no chronic medical illness at the age of two years.

Parental allergic history: Either one or both parents who self-reported having a physician-confirmed allergic disease, such as asthma, rhinoconjunctivitis, eczema, urticaria, or food allergies. 

### 2.5. Statistical Analysis 

The demographic data of the children were collected via questionnaires and analyzed. The numerical variables were presented as mean ± standard deviation or as frequencies and percentages. The associations between the categorical variables were assessed using the chi-square test. The continuous variables such as exclusive breastfeeding duration, age at solid food introduction, and allergic food diversity at 6 and 12 months of age, were analyzed using a one-way analysis of variance. 

Univariate and multivariate logistic regression analyses were used to examine whether exposure to infant formula, egg white, egg yolk, and peanuts (or nuts) in the first year of life was associated with IgE sensitization to any food or specific food in the BioIC test at 12 months of age. The following factors were considered as potential confounding variables that may contribute to the development of an allergy or be associated with infant feeding practices: gestational age ≤ 36 weeks; maternal and paternal allergy histories; maternal education; number of siblings; duration of exclusive breastfeeding; age at solid food introduction; allergenic food introduction (fruits, egg white, egg yolk, fish, shellfish, and peanuts); and any antipyretics or probiotic exposure during the first year of life. Odds ratios (ORs) and adjusted ORs (aORs) with 95% confidence intervals (CIs) are reported. The statistical significance of the regression analysis was set at *p* < 0.05. For each significant result in the regression analysis, the Bonferroni method was applied to correct the significance level of multiple comparisons (infant formula, egg white, egg yolk, and peanuts). A corrected *p*-value < 0.0125 was considered significant.

Logistic regression analyses were also performed for the AD outcomes. A univariate analysis was used to determine the relationships between allergenic food introduction, clinical variables, and physician-diagnosed AD by 2 years of age. The variables associated with physician-diagnosed AD by 2 years of age (*p* < 0.05) were entered into the multivariate logistic regression model to investigate the independent variables related to physician-confirmed AD by 2 years of age. Next, a stratified analysis regarding the parental history of allergy was performed to examine whether the association between egg introduction and AD varied with different genetic risks for allergy. The Bonferroni correction was additionally performed because the comparisons were multiple (both, one, or none of the parents with allergic histories). A corrected *p*-value < 0.017 was considered significant. Statistical analyses were performed using the SPSS software (version 26.0; IBM Corp., Armonk, NY, USA). The statistical significance was set at *p* < 0.05.

## 3. Results

### 3.1. Baseline Characteristics

A total of 501 children were included in the study. By the age of 2 years, 146 (29.1%) children had physician-diagnosed AD, and 309 (61.7%) children had a parental allergic history. The participants’ demographic characteristics are shown in Table 1. There were no significant differences in sex, delivery mode, maternal education, parental allergic history, and environmental risk factors between the children who developed AD and those who did not by 2 years of age. 

Allergen-specific IgE tests were performed in 310 (61.8%) infants at 12 months of age. Sensitization to peanuts was observed in 13.5% of the infants, egg yolk in 12.3%, cow’s milk in 10.6%, egg white in 9.7%, cheese in 5.5%, salmon in 5.5%, garlic in 5.4%, shrimp in 3.4%, wheat in 3.4%, soybean in 3.4%, potato in 3.4%, goat’s milk in 3.1%, codfish in 3.1%, almond in 2.8%, crab in 2.1%, kiwi in 0.6%, baker’s yeast in 0.3%, and blue mussel, carrot, or tomato in 0% of the infants. The number of children who were IgE-sensitized to at least one food allergen was higher in the AD than in the non-allergic group (47.5% vs. 22.7%, *p* < 0.001) (Table 1). 

The examination of infant feeding practices revealed a high variation in exclusive breastfeeding duration (mean: 4.00 ± 4.82 months). Although 86.7% of participants started eating solid foods between 4 and 5 months of age (mean: 4.46 ± 0.76 months), the timing of allergenic food introduction was variable. By the age of 1 year, the following had not been introduced to the infants: peanuts (*n* = 415, 82.8%), shellfish (*n* = 382, 76.2%), egg white (*n* = 165, 32.9%), egg yolk (*n* = 88, 17.6%), fish (*n* = 42, 8.4%), and fruits (*n* = 10, 2.0%). 

### 3.2. Intergroup Comparison of Allergenic Foods Introduction 

We observed a significant difference in the timing of egg white and egg yolk introduction between the children who developed AD and those who did not by 2 years of age (*p* = 0.001 and 0.006, respectively) (Table 1). In contrast, there was no significant difference in the exclusive breastfeeding duration, the timing of fruit, fish, shellfish, or peanut introduction, and the allergenic food diversity during the first year between AD and non-allergic children (Table 1). 

Additionally, we found that the participants who had both parents with allergic histories had been introduced to fewer allergenic food items in their diets compared with those who had one or no parent with an allergic history (3.52 ± 1.27 items vs. 3.82 ± 1.23 items vs. 3.90 ± 1.21 items, *p* = 0.039). The examination of the timing of egg white and yolk introduction showed that allergic parents tended to delay the introduction of egg white into infant diets, whereas the timing of egg yolk introduction was similar between allergic and non-allergic parents (egg white introduction by the age of 12 months: 63.7% vs. 72.3%, *p* = 0.057; egg yolk introduction by the age of 12 months: 83.4% vs. 80.9%, *p* = 0.542). Furthermore, 95 of 310 (30.6%) participants with IgE-sensitization to at least one food allergen tended to have a longer exclusive breastfeeding duration (5.39 ± 5.16 months vs. 3.83 ± 4.70 months, *p* = 0.014), a later introduction of solid foods (4.62 ± 0.82 months vs. 4.42 ± 0.71 months, *p* = 0.032), and less allergenic food diversity by the age of 1 year (3.51 ± 1.22 items vs. 3.87 ± 1.18 months, *p* = 0.024). 

### 3.3. Association between the Introduction of Allergenic Foods and Food Sensitization

The results of the unadjusted and adjusted analyses are shown in Table 2. Our data revealed that when compared to the reference group, infants below the age of 6 months who received any formula milk (mixed or exclusive) and were introduced to egg white, egg yolk, and peanuts from the age of 6–12 months had a decreased likelihood of developing any food (OR = 0.45–0.65) or specific food sensitization (OR = 0.19–0.65) (Table 2). Among these, the participants who were given egg whites during the first year of life were observed to have a significantly decreased risk of food sensitization (OR (95% CI) = 0.45 (0.26–0.76), *p* = 0.003; Bonferroni-corrected *p* < 0.0125), specifically egg white sensitization (0.19 (0.08–0.45), *p* < 0.001; Bonferroni-corrected *p* < 0.0125) at 12 months of age. In addition, the prevalence of cow’s milk sensitization was significantly decreased in infants who received any formula milk at <6 months of age (0.27 (0.11–0.69), *p* = 0.006; Bonferroni-corrected *p* < 0.0125). 

In the multivariable analyses, a similar effect was observed in the egg white introduction group in that the sensitization to any food or specific food was decreased after adjusting for potential confounding factors (aOR (95% CI) = 0.45 (0.23–0.89), *p* = 0.022 and (0.18 (0.06–0.51), *p* = 0.001, respectively). Nonetheless, this effect was not seen in the infants given early formula feeding (aOR (95% CI) = 1.68 (0.40–7.02) and 1.58 (0.25–10.16), *p* > 0.05) (Table 2). After Bonferroni’s adjustment, compared with the infants not introduced to egg white, the infants with egg white introduction showed a lower risk of egg white sensitization (corrected *p* < 0.0125) but did not significantly differ in terms of any food sensitization (corrected *p* > 0.0125). 

### 3.4. Identification of Independent Variables Related to Physician-Confirmed Atopic Dermatitis by 2 Years of Age

As shown in Table 3, a univariate analysis was first used to determine the relationships between the introduction of allergenic foods, clinical variables, and physician-diagnosed AD by 2-years-of-age. Our data revealed that a parental history of allergy and any antipyretics exposure during the first year after birth were positively associated (OR = 1.40 and 1.53, respectively), whereas egg white and yolk introduction (OR = 0.58 and 0.55, respectively) ≤12 months old were inversely associated with physician-diagnosed AD by the age of 2 years (*p* < 0.05). Although not significant, a trend suggested that exposure to a diversity of allergenic foods by the age of 12 months may reduce the risk of AD (OR = 0.87, *p* = 0.076). Next, the variables associated with physician-diagnosed AD by 2 years of age with values of *p* < 0.05 were entered into the multivariable logistic regression model to investigate the independent variables related to physician-confirmed AD by 2 years of age. In the results, egg white (aOR (95% CI) = 0.44 (0.24–0.82)) and egg yolk introduction (0.51 (0.27–0.97)) by the age of 12 months and a parental history of allergy (1.31 [1.01–1.70]) were significantly associated with AD by 2 years-of age (*p* < 0.05) (Table 3). 

### 3.5. Association between Egg White and Yolk Introduction and Physician-Confirmed AD by 2 Years of Age with Regard to Parental History of Allergy

Next, a stratified analysis regarding the parental history of allergy was performed to examine whether the association between egg introduction and AD varied with different genetic risks for allergy. Our data (Table 4) revealed that when compared with infants not introduced to eggs, infants who had been introduced to an entire egg (egg white and yolk) showed a negative association with physician-diagnosed AD by the age of 2 years, regardless of the parents’ allergic disease status (ORs = 0.34–0.65). These results were similar when adjusting for the potential confounding variables (aOR= 0.10–0.42), and the correlation was statistically significant among those children who had a history of allergy in both parents (aOR (95% CI) = 0.10 (0.02–0.59), *p* = 0.011) (Table 4). The result remained significant via the Bonferroni correction (corrected *p* < 0.017). 

For children who had both or either one of their parents with allergic histories, the introduction of either egg white or yolk may reduce the risk of physician-diagnosed AD by the age of 2 years. However, both the unadjusted and adjusted analyses showed there was no significant difference between the study groups of egg white or yolk introduction and egg avoidance (ORs = 0.78 and 0.69; adjusted ORs= 0.72 and 0.82, *p* > 0.05) (Table 4). 

## 4. Discussion 

The results from this prospective birth cohort study found that infants who had never been introduced to egg whites or yolks during the first year of life and whose parents had allergic diseases were more likely to develop physician-diagnosed AD by 2 years of age. Consequently, stratified analyses revealed that the introduction of both egg whites and yolks was negatively associated with AD by the age of 2 years when compared with the egg avoidance group, regardless of infant risk. The preventive effect of introduction to egg white and yolk was more apparent among those children where both parents had an allergic history. Taken together, our results suggest that egg white and yolk introduction may have immunomodulatory effects relevant to AD development in early childhood, which may be particularly important for infants where both parents have allergies.

Atopic dermatitis is a complex disease that may be triggered by environmental factors in genetically susceptible individuals [20]. Recently, accumulating evidence has suggested that the initiation of sensitization to allergens, including foods, occurs through a skin barrier impairment. An immune dysfunction is thought to exacerbate skin inflammation and form a vicious cycle [3,4,5]. Early nutrition influences immunological responses in the infant gut [21]. Our findings are consistent with the dual-allergen exposure hypothesis [3] that early exposure to food allergens may promote the maturation of the mucosal immune system, thereby inducing an immune tolerance network [21], which may reduce the inflammatory cascade of events that result in epicutaneous allergen sensitization and AD development in early childhood. 

In the past decade, several studies have demonstrated the induction of oral tolerance to allergenic foods in high-risk children [13,22]. For example, Palmer et al. reported that regular oral egg exposure from 4 to 8 months of age in infants with eczema can achieve the induction of immune tolerance pathways and a reduction in egg allergy incidence [23]. According to the enquiring about tolerance (EAT) study, introducing six allergenic foods to the diets of infants after 6 months of age among those with a sensitization to eggs at enrolment would lessen the incidence of egg allergy [24]. Regarding the timing of egg exposure for infants at high risk of developing an allergy, our results showed that the introduction of egg whites and yolks between 4 and 12 months of age was linked to a lower risk of physician-diagnosed AD by the age of 2 years. As only a few infants had been introduced to egg white (4.6%) or egg yolk (27.1%) before 6 months of age, the optimal timing for egg exposure to prevent AD remains inconclusive in our study. Similar to our findings, some studies have reported that introducing eggs at the age of 4 months is associated with a lower risk of egg sensitization and egg allergy [13,22,23,25,26], or indicated that delaying the introduction of eggs does not seem to be beneficial for AD [27,28,29]. Nonetheless, other studies considered that there is insufficient evidence to determine the relationship between the timing of egg introduction and AD [7,13,30]. The controversial results may be explained by the differences in races, cultural practices of infant feeding, and heterogeneity between the studies, including the risk characteristics of the study population and varied diagnostic methods and follow-up periods for AD [6,7,13,30]. Furthermore, scientific evidence has shown that the changes in specific IgE to egg white protein, rather than egg yolk protein, can be useful biomarkers for predicting persistent and subsequent tolerant egg-allergic children [31,32], implying that egg white and yolk have different allergenic properties [16]. Clinical trials have often examined egg protein exposure in various ways (e.g., pasteurized (raw)) egg white/whole egg powder, or whole cooked egg) [7,22,23,26,33], making it difficult to determine their relative involvement and effects on atopic outcomes. Additional well-designed prospective and randomized interventional studies are needed to clarify this issue. 

Consistent with international guidelines, the Taiwan Pediatric Association suggest that complementary foods should be introduced to infant diets at around 4–6 months. However, no clear recommendations are officially provided about the timing and method of the introduction of commonly allergenic foods into an infant’s diet. It is widely believed that prolonged breastfeeding and delaying the introduction of allergenic foods is beneficial regarding atopic diseases. In recent years, although a literature review has recommended exclusive breastfeeding for the first 3–4 months to decrease the incidence of eczema in the first 2 years of life, it remains unclear whether prolonged exclusive breastfeeding protects against allergic diseases [12,34]. There is evidence that food diversity in early life can reduce food sensitization and allergic asthma [3,35]. Our results, which recorded the allergen-specific IgE test results of 310 infants, failed to support the hypothesis that a longer duration of exclusive breastfeeding and the later introduction of specific foods conferred protection against the development of food sensitization at 12 months of age. These results imply that introducing various complementary foods earlier, including those regarded as allergenic, alongside breast milk feeding, rather than prolonged and exclusive breastfeeding with the later introduction of complementary foods, may be more important for reducing the prevalence of food sensitization in young children [34]. 

The strength of this study was its design. We explored the association between the introduction of egg white and egg yolk separately and in combination, and with the risk of food sensitization and AD in early childhood, which was different from other pediatric studies examining the whole-egg effect [6,7,13]. In addition, detailed information about feeding practices, allergenic food introduction, and the atopic manifestations of the participants were obtained on a regular basis, ensuring that recall bias was minimized. Furthermore, considering that parents’ perception and family history of allergy might affect the timing and types of allergenic food introduction, as well as the risk of developing AD in early childhood, we excluded those who had relapsing skin rashes or AD before introducing allergenic foods, performed a stratification analysis based on the parental history of allergy, and adjusted for potential confounding variables to reduce the effect of reverse causality. Given that such data are rarely reported in Asian children, the results of this study may provide a point of view to Asian parents who experience anxiety regarding their child’s dietary choices and subsequent allergic risk, particularly if both parents have a history of allergy.

This study has several limitations. The major limitation was that all the participants were enrolled from a single hospital, and 30.9% of the children who were either lost to follow-up or whose parents did not provide detailed dietary information during the first year were excluded from the study, resulting in the sample size for positive food sensitization, parental history of allergies, or AD being relatively small. Additionally, 38.1% of the enrolled patients did not complete the allergen-specific IgE test at the age of 1 year, indicating that selection bias cannot be ruled out. Another limitation is that we did not assess the frequency effects of egg yolk and white introduced to infant diets and the age when the children started consuming whole eggs, as well as the mixed effects of exposure to multiple allergenic foods. Although a multivariate logistic analysis and the Bonferroni test were performed to correct for confounding factors and adjust for multiple comparisons, it is difficult to clarify whether cross-interaction between different food components influences the risk of food sensitization and AD in observational studies. Thus, our data should be interpreted with caution. More studies comparing egg white, egg yolk, and whole egg introduction may yield further insights into the potential effects on childhood allergic diseases [16].

## 5. Conclusions 

In this study, our data revealed that a parental history of allergy was positively associated with physician-diagnosed AD by the age of two years, while the introduction of egg whites and yolks to infant diets was negatively associated. In addition, compared with the children who had either one or no parents with allergies, we found that in the children where both parents had a history of allergy there was a more apparent effect of introducing both egg whites and yolks on reducing AD development. 

Based on our results, we suggest that the introduction of egg white and yolk to infant diets may induce an oral tolerance effect and may be a modifiable factor for reducing the risk of physician-diagnosed AD by the age of 2 years. The preventive effect of egg consumption may be particularly important in infants with a parental history of allergy. Large-scale longitudinal studies are required to confirm the findings of this study.

## Figures and Tables

**Table 1:** Demographic data and infant feeding characteristics of the study participants.

Characteristics	Children Who Developed Atopic Dermatitis by the Age of 2 Years (AD Children) (*N* = 146)	Non-Allergic Children (*N* = 355)	*p*-Value
**Neonatal and environmental factors**			
Sex of infants, male (%)	85 (58.2)	185 (52.1)	0.188
Gestational age (week)	38.1 ± 1.3	38.5 ± 1.2	0.158
Cesarean section, *n* (%)	50 (34.2)	121 (34.1)	0.999
Birth body weight (kg)	3.1 ± 0.4	3.1 ± 0.4	0.352
Sibling 0 1 ≥2	74 (50.7) 53 (36.3) 19 (13.0)	196 (55.2) 115 (32.4) 44 (12.3)	0.639
Environmental tobacco smoking, *n* (%)	72 (49.3)	170 (47.9)	0.844
Household pets during the first year, *n* (%)	40 (27.4)	107 (30.1)	0.590
Medical exposure histories during the first year, *n* (%) Antipyretics Probiotics	109 (74.7) 85 (58.2)	236 (66.5) 186 (52.2)	0.052 0.254
**Parental factors**			
Maternal age (y)	31.1 ± 4.5	31.2 ± 4.7	0.889
Maternal education level High school or below, *n* (%) College or above, *n* (%)	32 (22.0) 114 (78.0)	84 (23.7) 271 (76.3)	0.727
Parental history of allergy Only maternal allergy, *n* (%) Only paternal allergy, *n* (%) Both maternal and paternal allergy, *n* (%)	28(19.2) 29 (19.9) 39 (26.7)	72 (20.2) 77 (21.7) 64 (18.0)	0.161
**Infant feeding practices**			
Exclusive breastfeeding duration, months <6 months old, *n* (%) ≥6–12 months old, *n* (%) Never ^a^, *n* (%)	4.04 ± 4.75 29 (19.9) 42 (28.8) 75 (51.3)	3.98 ± 4.86 61(17.2) 101(28.5) 193(54.4)	0.892 0.744
Age at which solid foods were introduced ≥4–5 months old, *n* (%) ≥6 months old, *n* (%)	4.50 ± 0.75 125 (85.6) 21 (14.4)	4.46 ± 0.76 309 (87.1) 46 (13.0)	0.901 0.667
Timing of egg white introduction ≥4–5 months, *n* (%) ≥6–12 months, *n* (%) Never ^b^, *n* (%)	7 (5.6) 73 (54.2) 66 (40.1)	16 (4.5) 240 (67.6) 99 (27.9)	0.001 *
Timing of egg yolk introduction ≥4–5 months, *n* (%) ≥6–12 months, *n* (%) Never ^b^, *n* (%)	27 (18.5) 83 (56.8) 36 (24.7)	51 (14.4) 252 (71.0) 52 (14.6)	0.006 *
Timing of fruit introduction ≥4–5 months, *n* (%) ≥6–12 months, *n* (%) Never ^b^, *n* (%)	105 (71.9) 38 (26.0) 3 (2.1)	255 (71.8) 93 (26.2) 7 (2.0)	0.998
Timing of fish introduction ≥4–5 months, *n* (%) ≥6–12 months, *n* (%) Never ^b^, *n* (%)	20 (13.7) 112 (76.7) 14 (9.6)	54 (15.2) 273 (76.9) 28 (7.9)	0.772
Timing of shellfish introduction ≥6–12 months, *n* (%) Never ^b^, *n* (%)	39 (26.7) 107 (73.3)	80 (22.5) 275 (77.5)	0.355
Timing of peanut introduction ≥6–12 months, *n* (%) Never ^b^, *n* (%)	26 (17.8) 120 (82.3)	60 (16.9) 295 (83.1)	0.796
Allergenic foods diversity ^c^ At age 6 months, items At age 12 months, items	1.12 ± 0.97 3.65 ± 1.34	1.09 ± 1.01 3.86 ± 1.17	0.702 0.075
**Allergen-specific IgE at age 1 year, *n* (%)**	99 (67.8)	211 (59.4)	0.086
Any food allergen sensitization, *n* (%)	47 (47.5)	48 (22.7)	<0.001 *

*p* value based on the chi-square test and one-way analysis of variance test. Statistically significant: * *p* < 0.05. (a) Infants who were exclusively fed with formula or who were partially breastfed. (b) A food item that was never introduced during the first year of life. (c) Allergenic foods’ diversity was calculated by means of six food items (fruits, egg white, egg yolk, fish, shellfish, and peanuts) in the study.

**Table 2:** Allergenic food introduction during the first year and the prevalence of food sensitization at the age of 12 months.

		Any Food Sensitization (*N* = 95)				Specific Food Sensitization			
		OR (95% CI)	*p* Value	Adjusted OR (95% CI)	*p* Value	OR (95% CI)	*p* Value	Adjusted OR (95% CI)	*p* Value
Exposure to infant formula (Reference: no formula feeding)						IgE to cow’s milk (*N* = 33)			
<6 months old		0.61 (0.30–1.23)	0.166	1.68 (0.40–7.02)	0.476	0.27 (0.11–0.69)	0.006*	1.58 (0.25–10.16)	0.627
≥6–12 months old		1.28 (0.54–3.03)	0.570	2.03 (0.72–5.76)	0.182	1.11 (0.40–3.08)	0.839	2.56 (0.70–9.36)	0.154
Egg white introduction (Reference: egg white avoidance)						IgE to egg white (*N* = 30)			
≤12 months old		0.45 (0.26–0.76)	0.003 *	0.45 (0.23–0.89)	0.022 *	0.19 (0.08–0.45)	<0.001 *	0.18 (0.06–0.51)	0.001 *
Egg yolk introduction (Reference: egg yolk avoidance)						IgE to egg yolk (*N* = 38)			
≤12 months old		0.65 (0.34–1.23)	0.188	0.82 (0.37–1.81)	0.621	0.65 (0.27–1.53)	0.322	0.63 (0.21–1.89)	0.407
Peanuts (or nuts) introduction (Reference: peanuts (or nuts) avoidance)						IgE to peanuts (*N* = 42)			
≤12 months old		0.60 (0.27–1.33)	0.210	0.50 (0.19–1.33)	0.166	0.49 (0.14–1.66)	0.250	0.36 (0.07–1.77)	0.207

OR: odds ratio; CI: confidence interval; OR adjusted for gestational ages ≤ 36 weeks, parental allergy (both, either one or none), maternal education, sibling numbers, exclusive breastfeeding duration, age at solid food introduction, allergenic food introduction (fruits, egg white, egg yolk, fish, shellfish, and peanuts), and any antipyretics or probiotics exposure by the age of 12 months. Statistically significant: * *p* < 0.05.

**Table 3:** Association between physician-diagnosed atopic dermatitis by the age of 2 years, infant feeding practices, and the clinical variables for allergy.

	Univariate Analysis		Multivariable Analysis	
	OR (95% CI)	*p* Value	Adjusted OR (95% CI)	*p* Value
**Infant feeding practices**				
Exclusive breastfeeding duration by the age of 12 months (months)	1.00 (0.96–1.04)	0.892		
Age at which solid foods introduction (months)	1.02 (0.80–1.29)	0.901		
Exposure to infant formula < the age of 6 months	1.08 (0.71–1.64)	0.715		
Egg white introduction by the age of 12 months	0.58 (0.39–0.86)	0.006 *	0.44 (0.24–0.82)	0.009 *
Egg yolk introduction by the age of 12 months	0.55 (0.34–0.88)	0.013 *	0.51 (0.27–0.97)	0.040 *
Peanuts (or nuts) introduction by the age of 12 months	1.03 (0.63–1.71)	0.898		
Allergenic foods diversity by the age of 12 months	0.87 (0.63–1.71)	0.076		
**Clinical variables**				
Gestational ages ≤ 36 weeks	1.34 (0.70–2.56)	0.381		
Mode of delivery (cesarean section)	1.01 (0.69–1.49)	0.958		
Parental history of allergy (both, either one, or none)	1.40 (1.09–1.79)	0.008 *	1.31 (1.01–1.70)	0.046 *
Household pets during the first year	0.89 (0.59–1.35)	0.579		
Environmental tobacco smoking during the first year	1.05 (0.72–1.52)	0.818		
Antipyretics exposure history during the first year	1.53 (1.01–2.31)	0.046 *	1.54 (0.99–2.39)	0.057

OR: odds ratio; CI: confidence interval. Statistically significant: * *p* < 0.05.

**Table 4:** Egg white and yolk introduction during the first year and the prevalence of physician-diagnosed atopic dermatitis by the age of 2 years, stratified by parental history of allergy.

	Both Parents Had an Allergy (*N* = 39)		Either Parent Had an Allergy (*N* = 57)		Neither Parent Had an Allergy (*N* = 50)	
	OR (95% CI)	Adjusted OR (95% CI)	OR (95% CI)	Adjusted OR (95% CI)	OR (95% CI)	Adjusted OR (95% CI)
Egg white and yolk introduction (Reference: egg avoidance)						
≤12 months old	0.34 (0.12–1.01)	0.10 (0.02–0.59)	0.50 (0.21–1.17)	0.42 (0.14–1.24)	0.65 (0.24–1.77)	0.34 (0.10–1.18)
*p* value	0.052	0.011 *#	0.111	0.115	0.400	0.090
Either egg white or yolk introduction (Reference: egg avoidance)						
≤12 months old	0.78 (0.24–2.50)	0.72 (0.11–4.82)	0.69 (0.26–1.83)	0.82 (0.24–2.83)	1.17 (0.38–3.58)	0.63 (0.13–3.03)
*p* value	0.675	0.734	0.455	0.149	0.788	0.560

OR: odds ratio; CI: confidence interval; OR adjusted for gestational ages ≤ 36 weeks, maternal education, sibling numbers, exclusive breastfeeding duration, age at solid food introduction, allergenic food introduction (fruits, egg white, egg yolk, fish, shellfish, and peanuts), and any antipyretics or probiotics exposure by the age of 12 months. Statistically significant in logistic regression analysis: * *p* < 0.05. Statistically significant via Bonferroni correction: # Corrected *p* < 0.017.

## Data Availability

The data will not be openly available because they contain the personal information of the participants. However, the datasets generated for this study are available from the corresponding author upon reasonable request.

## References

[1] Tsakok T., Marrs T., Mohsin M., Baron S., du Toit G., Till S., Flohr C. (2016). Does atopic dermatitis cause food allergy? A systematic review. J. Allergy Clin. Immunol..

[2] Domínguez O., Plaza A.M., Alvaro M. (2020). Relationship Between Atopic Dermatitis and Food Allergy. Curr. Pediatr. Rev..

[3] Brough H.A., Nadeau K.C., Sindher S.B., Alkotob S.S., Chan S., Bahnson H.T., Leung D.Y.M., Lack G. (2020). Epicutaneous sensitization in the development of food allergy: What is the evidence and how can this be prevented?. Allergy.

[4] Allenova A., Darlenski R. (2023). The hen and the egg question in atopic dermatitis: Allergy or eczema comes first. Asthma Res. Pract..

[5] Tham E.H., Leung D.Y. (2019). Mechanisms by Which Atopic Dermatitis Predisposes to Food Allergy and the Atopic March. Allergy Asthma Immunol. Res..

[6] Burgess J.A., Dharmage S.C., Allen K., Koplin J., Garcia-Larsen V., Boyle R., Waidyatillake N., Lodge C.J. (2019). Age at introduction to complementary solid food and food allergy and sensitization: A systematic review and meta-analysis. Clin. Exp Allergy.

[7] Obbagy J.E., English L.K., Wong Y.P., Butte N.F., Dewey K.G., Fleischer D.M., Fox M.K., Greer F.R., Krebs N.F., Scanlon K.S. (2019). Complementary feeding and food allergy, atopic dermatitis/eczema, asthma, and allergic rhinitis: A systematic review. Am. J. Clin. Nutr..

[8] Caffarelli C., Di Mauro D., Mastrorilli C., Bottau P., Cipriani F., Ricci G. (2018). Solid Food Introduction and the Development of Food Allergies. Nutrients.

[9] Tran M.M., Lefebvre D.L., Dai D., Dharma C., Subbarao P., Lou W., Azad M.B., Becker A.B., Mandhane P.J., Turvey S.E. (2017). Timing of food introduction and development of food sensitization in a prospective birth cohort. Pediatr. Allergy Immunol..

[10] Halken S., Muraro A., de Silva D., Khaleva E., Angier E., Arasi S., Arshad H., Bahnson H.T., Beyer K., Boyle R. (2021). European Academy of Allergy and Clinical Immunology Food Allergy and Anaphylaxis Guidelines Group. EAACI guideline: Preventing the development of food allergy in infants and young children (2020 update). Pediatr. Allergy Immunol..

[11] Fewtrell M., Bronsky J., Campoy C., Domellöf M., Embleton N., Fidler Mis N., Hojsak I., Hulst J.M., Indrio F., Lapillonne A. (2017). Complementary Feeding: A Position Paper by the European Society for Paediatric Gastroenterology, Hepatology, and Nutrition (ESPGHAN) Committee on Nutrition. J. Pediatr. Gastroenterol. Nutr..

[12] Greer F.R., Sicherer S.H., Burks A.W. (2019). Committee on Nutrition, & Section on Allergy and Immunology. The Effects of Early Nutritional Interventions on the Development of Atopic Disease in Infants and Children: The Role of Maternal Dietary Restriction, Breastfeeding, Hydrolyzed Formulas, and Timing of Introduction of Allergenic Complementary Foods. Pediatrics.

[13] Ierodiakonou D., Garcia-Larsen V., Logan A., Groome A., Cunha S., Chivinge J., Robinson Z., Geoghegan N., Jarrold K., Reeves T. (2016). Timing of Allergenic Food Introduction to the Infant Diet and Risk of Allergic or Autoimmune Disease: A Systematic Review and Meta-analysis. JAMA.

[14] Cook E.J., Powell F.C., Ali N., Penn-Jones C., Ochieng B., Randhawa G. (2021). Parents’ experiences of complementary feeding among a United Kingdom culturally diverse and deprived community. Matern. Child Nutr..

[15] Lee A.J., Thalayasingam M., Lee B.W. (2013). Food allergy in Asia: How does it compare?. Asia Pac. Allergy.

[16] Dosanjh A. (2017). Egg introduction: Differential allergic responses. J. Asthma Allergy.

[17] Tang R., Wang Z.X., Ji C.M., Leung P.S.C., Woo E., Chang C., Wang M., Liu B., Wei J.F., Sun J.L. (2019). Regional Differences in Food Allergies. Clin. Rev. Allergy Immunol..

[18] Hua M.C., Yao T.C., Chen C.C., Tsai M.H., Liao S.L., Lai S.H., Chiu C.Y., Su K.W., Yeh K.W., Huang J.L. (2017). Introduction of various allergenic foods during infancy reduces risk of IgE sensitization at 12 months of age: A birth cohort study. Pediatr. Res..

[19] Hua M.C., Su H.M., Kuo M.L., Chen C.C., Yao T.C., Tsai M.H., Liao S.L., Lai S.H., Chiu C.Y., Su K.W. (2019). Association of maternal allergy with human milk soluble CD14 and fatty acids, and early childhood atopic dermatitis. Pediatr. Allergy Immunol..

[20] Løset M., Brown S.J., Saunes M., Hveem K. (2019). Genetics of Atopic Dermatitis: From DNA Sequence to Clinical Relevance. Dermatology.

[21] Prescott S.L., Smith P., Tang M., Palmer D.J., Sinn J., Huntley S.J., Cormack B., Heine R.G., Gibson R.A., Makrides M. (2008). The importance of early complementary feeding in the development of oral tolerance: Concerns and controversies. Pediatr. Allergy Immunol..

[22] Perkin M.R., Logan K., Tseng A., Raji B., Ayis S., Peacock J., Brough H., Marrs T., Radulovic S., Craven J. (2016). Randomized Trial of Introduction of Allergenic Foods in Breast-Fed Infants. N. Engl. J. Med..

[23] Palmer D.J., Metcalfe J., Makrides M., Gold M.S., Quinn P., West C.E., Loh R., Prescott S.L. (2013). Early regular egg exposure in infants with eczema: A randomized controlled trial. J. Allergy Clin. Immunol..

[24] Perkin M.R., Logan K., Bahnson H.T., Marrs T., Radulovic S., Craven J., Flohr C., Mills E.N., Versteeg S.A., van Ree R. (2019). Enquiring About Tolerance (EAT) study team. Efficacy of the Enquiring About Tolerance (EAT) study among infants at high risk of developing food allergy. J. Allergy Clin. Immunol..

[25] Nwaru B.I., Erkkola M., Ahonen S., Kaila M., Haapala A.M., Kronberg-Kippilä C., Salmelin R., Veijola R., Ilonen J., Simell O. (2010). Age at the introduction of solid foods during the first year and allergic sensitization at age 5 years. Pediatrics.

[26] Wei-Liang Tan J., Valerio C., Barnes E.H., Turner P.J., Van Asperen P.A., Kakakios A.M., Campbell D.E., Beating Egg Allergy Trial (BEAT) Study Group (2017). A randomized trial of egg introduction from 4 months of age in infants at risk for egg allergy. J. Allergy Clin. Immunol..

[27] Filipiak B., Zutavern A., Koletzko S., von Berg A., Brockow I., Grübl A., Berdel D., Reinhardt D., Bauer C.P., Wichmann H.E. (2007). Solid food introduction in relation to eczema: Results from a four-year prospective birth cohort study. J. Pediatr..

[28] Roduit C., Frei R., Loss G., Büchele G., Weber J., Depner M., Loeliger S., Dalphin M.L., Roponen M., Hyvärinen A. (2012). Protection Against Allergy–Study in Rural Environments study group. Development of atopic dermatitis according to age of onset and association with early-life exposures. J. Allergy Clin. Immunol..

[29] Zutavern A., von Mutius E., Harris J., Mills P., Moffatt S., White C., Cullinan P. (2004). The introduction of solids in relation to asthma and eczema. Arch. Dis. Child..

[30] Niinivirta K., Isolauri E., Nermes M., Laitinen K. (2014). Timing of complementary feeding and the risk of atopic eczema. Acta Paediatr..

[31] Gradman J., Mortz C.G., Eller E., Bindslev-Jensen C. (2016). Relationship between specific IgE to egg components and natural history of egg allergy in Danish children. Pediatr. Allergy Immunol..

[32] Kim J.D., Kim S.Y., Kwak E.J., Sol I.S., Kim M.J., Kim Y.H., Kim K.W., Sohn M.H. (2019). Reduction Rate of Specific IgE Level as a Predictor of Persistent Egg Allergy in Children. Allergy Asthma Immunol. Res..

[33] Natsume O., Kabashima S., Nakazato J., Yamamoto-Hanada K., Narita M., Kondo M., Saito M., Kishino A., Takimoto T., Inoue E. (2017). Two-step egg introduction for prevention of egg allergy in high-risk infants with eczema (PETIT): A randomized, double-blind, placebo-controlled trial. Lancet.

[34] Kramer M.S., Kakuma R. (2012). Optimal duration of exclusive breastfeeding. Cochrane Database Syst. Rev..

[35] Venter C., Greenhawt M., Meyer R.W., Agostoni C., Reese I., du Toit G., Feeney M., Maslin K., Nwaru B.I., Roduit C. (2020). EAACI position paper on diet diversity in pregnancy, infancy and childhood: Novel concepts and implications for studies in allergy and asthma. Allergy.

